# Uptake and metabolization of four sartan drugs by eight different plants: Targeted and untargeted analyses by HPLC‐drift‐tube‐ion‐mobility quadrupole time‐of‐flight mass spectrometry

**DOI:** 10.1002/elps.202300134

**Published:** 2023-11-09

**Authors:** Laura Zellner, Thomas Schiefer, Markus Himmelsbach, Franz Mlynek, Christian W. Klampfl

**Affiliations:** ^1^ Institute of Analytical and General Chemistry Johannes Kepler University Linz Austria

**Keywords:** drift‐tube ion‐mobility–mass spectrometry, environmental analysis, plant metabolism, plant uptake, sartans

## Abstract

In this study, we investigated the uptake and metabolization of four drugs (plus the associated prodrugs) from the sartan family by eight edible plants. Growing the plants hydroponically in a medium containing the respective drug, more than 40 phases I and II metabolites derived from the four sartan drugs could be tentatively identified. To demonstrate the suitability of the proposed analytical approach for actual environmental samples, garden cress (*Lepidium sativum*) selected as a model plant was grown in water drawn from the effluent of two local wastewater treatment plants. Thereby, three of the sartans, namely, olmesartan, candesartan, and valsartan, could be found in the plant extracts at concentrations of 3.1, 10.4, and 14.4 ng g^−1^, respectively. Additionally, for candesartan and valsartan, a glycosylated transformation product could be detected. In order to extend the present (targeted) workflow also toward the analysis of unknown transformation products (i.e., those not listed in the custom‐made database used for this research), a nontargeted approach for the analysis of plant extracts with respect to the presence of drug‐related metabolites was developed. Comparison of the targeted and the nontargeted workflows led to the finding of two additional, so far unidentified, transformation products originating from azilsartan.

**Abbreviations**
API: active pharmaceutical ingredient
AZ: azilsartan
AZ‐MD: azilsartan medoxomil
CAN: candesartan
CCS: collision cross‐section
CDC: candesartan cilexetil
DT–IM QTOF–MS: drift tube ion mobility quadrupole time‐of flight mass spectrometry
FC: fold changes
HR‐MS/MS: high‐resolution multiple‐stage mass spectrometry
IMS‐HR‐MS/MS: ion mobility‐high‐resolution multiple‐stage mass spectrometry
OL: olmesartan
OL‐MD: olmesartan medoxomil
PPCP: pharmaceuticals and personal care products
VAL: valsartan
WWTP: wastewater treatment plants

## 1 INTRODUCTION

Research on the uptake, translocation, and biotransformation of xenobiotics by plants has been the subject of a number of studies published within the last decades (for some exemplary reviews, see Refs. [1, 2, 3, 4, 5, 6, 7]). Amongst others, one main reason triggering increased interest in this topic is the need for raising the output from agricultural production, allowing to feed a growing population worldwide. The struggle to reach this goal is unfortunately hampered by deteriorating conditions for growing edible plants in many regions of our planet, as climate change leads to steady growth in areas with substantial scarcity of water (whereby increased temperatures and less rainfall need to be compensated by irrigation of the fields). To overcome the latter problem, the use of reclaimed waters from wastewater treatment plants (WWTPs) for irrigation became more and more common in agriculture. Although these waters (either from industrial sites or municipalities) are treated in a multistage process, they still may include a range of contaminants in small concentrations. A prominent subgroup of these so‐called micropollutants are pharmaceuticals and personal care products (PPCPs) (for some relevant reviews, see Refs. [8, 9, 10]). Due to this water reuse [11, 12, 13], crops cultivated for consumption by humans or as a feed for livestock may come into contact with PPCPs, which are subsequently incorporated in the plant.

Investigations to study this subject may be solely focused on the uptake of these substances from water or soil (for some relevant reviews, see Refs. [1–4, 14, 15]) but also on the subsequent fate within the plant such as translocation to different plant parts or metabolization of the parent substance by the plant organism (for some relevant reviews, see Refs. [5, 6, 7]). For studying these processes, the growing of plants under hydroponic conditions (while introducing the contaminant of interest via the growing medium) can be regarded as a straightforward approach to gain the desired knowledge [3, 16]. Mostly, to facilitate the detection and (tentative) identification of the metabolites formed, the pharmaceuticals under investigation are added at much higher concentrations (mg L^−1^ range) compared to those commonly found in real environmental samples. Nevertheless, there are also studies where plants grown under hydroponic conditions or in the field were subjected to actual reclaimed waters. Although these studies were mostly directed at the issue of uptake, still drug metabolites formed within the plant could be detected in some cases [17, 18, 19]. Of course, under these circumstances, only the most abundant metabolites could be found at very low‐concentration levels. These low‐concentration levels and the fact that information‐rich detection is required to at least allow the tentative suggestion of metabolites originating from the parent drug either through phase I or phase II metabolization make high‐performance liquid chromatography (HPLC) coupled to high‐resolution multiple‐stage mass spectrometry (HR‐MS/MS) the most suitable tool for this challenge. Recently, the introduction of HR‐MS/MS instruments combined with ion mobility spectrometry (IMS) added a further parameter (the collision cross‐section [CCS]) to the set (retention time from chromatography, accurate mass, fragmentation in MS/MS) already employed for the characterization of the analytes under investigation.

In the present paper, we used HPLC coupled to drift tube (DT) IMS‐HR‐MS/MS to characterize extracts from eight different plants cultivated hydroponically, either in a growing medium spiked with four common pharmaceuticals from the sartan family or in actual waters collected from WWTP effluents. The extracts from these plants were searched for potential drug‐derived metabolites either by a fast screening approach recently published by our group [20] or by nontargeted analysis comparing the features from treated and non‐treated plants.

## 2 MATERIALS AND METHODS

### 2.1 Chemicals and materials

Azilsartan medoxomil (AZ‐MD, 20 mg, Edarbi, Takeda Ges.m.b.H), candesartan cilexetil (CDC, 32 mg, 1A Pharma GmbH), olmesartan medoxomil (OL‐MD, 10 mg, Olmetec, Daiichi Sankyo Austria GmbH), and valsartan (VAL, 80 mg, Diovan, Novartis Pharma GmbH) were purchased as pharmaceutical preparations in a local pharmacy. Stock solutions of the pharmaceuticals were prepared by suspending one tablet, respectively, in 10 mL MeOH, followed by ultrasonication (Elmasonic S 60 H, Elma) for 20 min. Thereby, solutions of 2000 mg L^−1^ AZ‐MD, 3200 mg L^−1^ CDC, 1000 mg L^−1^ OL‐MD, and 8000 mg L^−1^ VAL were obtained. For plant cultivation, 10 mg L^−1^ solutions for each pharmaceutical were prepared by further dilution in tap water.

To achieve full hydrolysis of the prodrugs for quantitative analysis, a dilution in 1 M NaOH was made. Complete hydrolysis occurred within 30 min and was verified using HPLC.

Acetonitrile and methanol were purchased from VWR (HPLC grade). Formic acid and hydrochloric acid (37%, analytical reagent grade) were acquired from Sigma‐Aldrich and Merck, respectively.

Ultrapure water was obtained from a Milli‐Q water purification system (Millipore).

Carrots (rote Riesen, *Daucus carota* ssp. *Sativus*) and garden cress (Cresto, *Lepidium sativum*) were purchased in a local garden shop (brand Kiepenkerl). Pea (Tiberius, *Pisum sativum*), maize (LG31272, *Zea mays*), sorghum (Armorik, *Sorghum bicolor* L.), triticale (Borowik, *Triticosecale neoblaringhemii*), rye (KWS Binntto, *Secale cereale*), and barley (Ernesta, *Hordeum vulgare*) were obtained from Raiffeisen Ware Austria AG (RWA).

### 2.2 Plant cultivation and treatment

All plants were hydroponically grown without any addition of nutrients. Approximately 5 g of cress seeds were immersed in 250 mL of tap water for 2 h. Further, they were distributed on the grid of a cultivation set and grown for 1 week in tap water on the lab bench. The tap water was then exchanged with a 10‐mg L^−1^ solution of the active pharmaceutical ingredient (API) and grown for another 7 days, whereby the medium was refilled to assure constant supply.

Maize and pea seeds were soaked in tap water overnight and germinated on a wet paper towel for 2 days in the dark. The germinated seeds were planted into beds of wetted iron‐on beads and grown for a few days. When they reached a certain height, they could be transferred into small Erlenmeyer flasks filled with 50 mL tap water or API solution and were grown for 1 week on the lab bench.

Rye, triticale, and barley seeds were soaked in tap water overnight and germinated on a wet paper towel for 2 days in the dark. The plantlets were transferred to petri dishes, containing a paper towel soaked in either tap water or API solution and were grown for 7 days on the lab bench.

Carrot and sorghum seeds were placed in petri dishes and germinated in the dark for 2 days with small amounts of tap water or API solution. The sprouts were further grown for 5 days on the lab bench, whereby the cultivation medium was supplied to the plantlets on daily basis.

### 2.3 Preparation of plant extracts

The plants were washed thoroughly with tap water and plotted dry with paper towels. The plants were separated either into roots and leaves or roots, stems, and leaves. About 1.5 g (0.5 g for cress) of the plant material (wet weight) were weighed into 15 mL centrifugation tubes, 3 mL (1 mL for cress) of extraction solvent (0.1 M HCl/ACN 2:1 v/v), as well as four steel balls were added. The samples were extracted for 15 min at 20 Hz using a swing mill (“Star Beater,” VWR) followed by centrifugation (“MegaStar” 1.6 R, VWR) at 4200 *g* for 8 min at 4°C. The supernatant was filtered through nylon syringe filters (CHROMAFIL, pore size 0.45 µm, *Ø* 15 mm, Macherey‐Nagel) into an HPLC glass vial and stored at −80°C until analysis.

### 2.4 HPLC–DT–IM–QTOF–MS and HPLC‐QqQ–MS/MS

For compound separation, a reversed‐phase (RP) HPLC (Agilent 1260, Agilent Technologies) was equipped with a Poroshell 120 EC‐C18 column (3 × 75 mm^2^, 2.7 µm, Agilent Technologies) guarded with a C18 guard column (4 × 3 mm^2^, 3 µm, Phenomenex). Mobile phase A consisted of water with 0.1% formic acid, mobile phase B of acetonitrile with 0.1% formic acid. The starting conditions were 90% A and 10% B, which was held for 0.5 min. Mobile phase B was then increased to 35% until minute 4.5 and finally to 95% B until minute 9. This was held for 3 min, and the column was re‐equilibrated for 6 min. The flow rate was set to 0.5 mL min^−1^, the column temperature was held at 30°C, and an injection volume of 20 µL was chosen.

For the quantification of the APIs in the cress samples treated with the WWTP effluent, an Agilent 6420 triple quadrupole QqQ‐MS/MS (Agilent Technologies) was used. The Agilent Dual Jet stream source was operated in positive mode, and the source parameters were as follows: The drying/sheath gas temperature was 300°C, the drying/sheath gas flow rate was 10 L min^−1^ (both nitrogen), the nebulizer pressure was 35 psi, the capillary voltage was 4000 V, and the nozzle voltage was 1000 V. The delta EMV (+) was 400, and the information regarding the transitions can be found in Table S1. Matrix‐matched calibration was used for quantification by preparing standards in a cress extract where plants were grown without analytes.

For the targeted and untargeted tentative metabolite identification, the HPLC system was coupled to a high‐resolution DT–IM–quadrupole time‐of‐flight (QTOF)–MS (Agilent 6560, Agilent Technologies) equipped with an Agilent Dual Jet Stream Electrospray Ionization source, which was operated in positive ion mode. The drying/sheath gas temperature was 275°C, the drying/sheath gas flow rate was 11 L min^−1^ (both nitrogen), the nebulizer pressure was 40 psi, the capillary voltage was 4000 V, the nozzle voltage was 1000 V, and the fragmentor voltage was 400 V.

The DT–IM–QTOF–MS was tuned in the “fragile ion” mode and operated with 5‐bit multiplexing. The settings were as follows: trap fill time was 1800 µs, trap release time was 250 µs, frame rate was 0.9 frames s^−1^, transient rate was 17 transients frame^−1^, and maximum drift time was 60 ms. Under advanced parameters following settings were set: DT entrance voltage was 1567 V, DT exit voltage was 217 V, rear funnel entrance was 210.5 V, and rear funnel exit was 38 V.

### 2.5 Data processing

Data evaluation/processing was done by using Agilent MassHunter Qualitative Analysis B.07.00, Agilent MassHunter Workstation Quantitative Analysis 10.1, PNNL PreProcessor 4.0 (2022.02.18), IM‐MS Browser 10.0.1, and Agilent Masshunter Workstation Mass Profiler 10.0.2.

The PNNL PreProcessor software was used for demultiplexing the IM data files. The data were calibrated with the recorded single‐field tune using IM‐MS browser. The drift times and the ^DT^CCS_N2_ were determined by performing a feature extraction using the Mass Profiler software. Following parameters were chosen: ion intensity ≥2500 counts, RT tolerance of 0.2%, drift time tolerance of 1.5%, mass tolerance of 5 ppm and *Q*‐score of 60.

### 2.6 Untargeted analysis—Plantomics

Five samples each for cress grown in tap water and in AZ‐MD solution were used for the untargeted workflow. For data processing, analysis, and visualization, a script compiled in Python (version 3.9) was used (libraries pandas 2.0.1, numpy 1.24.3, matplotlib 3.7.1, scipy 1.10.1, plotly 5.14.1, statsmodels 0.14.0, and seaborn 0.12.2). First, the data of the extracted features were preprocessed as follows: features showing equal intensity over all samples (background signals stemming from the solvent, the tap water, etc.) and features near the base line were excluded. Missing values were replaced by 1/5 of the minimal value of the respective feature, and the data were pareto scaled and transformed by cubic root.

Fold changes (FCs) and *p* values were computed for statistical analysis. FCs were calculated after the replacement of the missing values but prior to the remaining preprocessing. *p* Values were computed using a *t*‐test for two independent samples with the Benjamini Hochberg correction.

Hierarchically clustered heatmaps were created from the 30 most significant features (based on *t*‐tests) using Ward's method and an Euclidean distance metric. For the volcano plots, FCs were log_2_ and *p*‐values log_10_ transformed and thresholds were set to 2.0 and 0.05, respectively. The thereby found most promising upregulated features of the API‐treated samples were further analyzed by targeted MS/MS.

## 3 RESULTS AND DISCUSSION

### 3.1 Uptake and metabolization of valsartan, candesartan, azilsartan, and olmesartan

**FIGURE 1:**
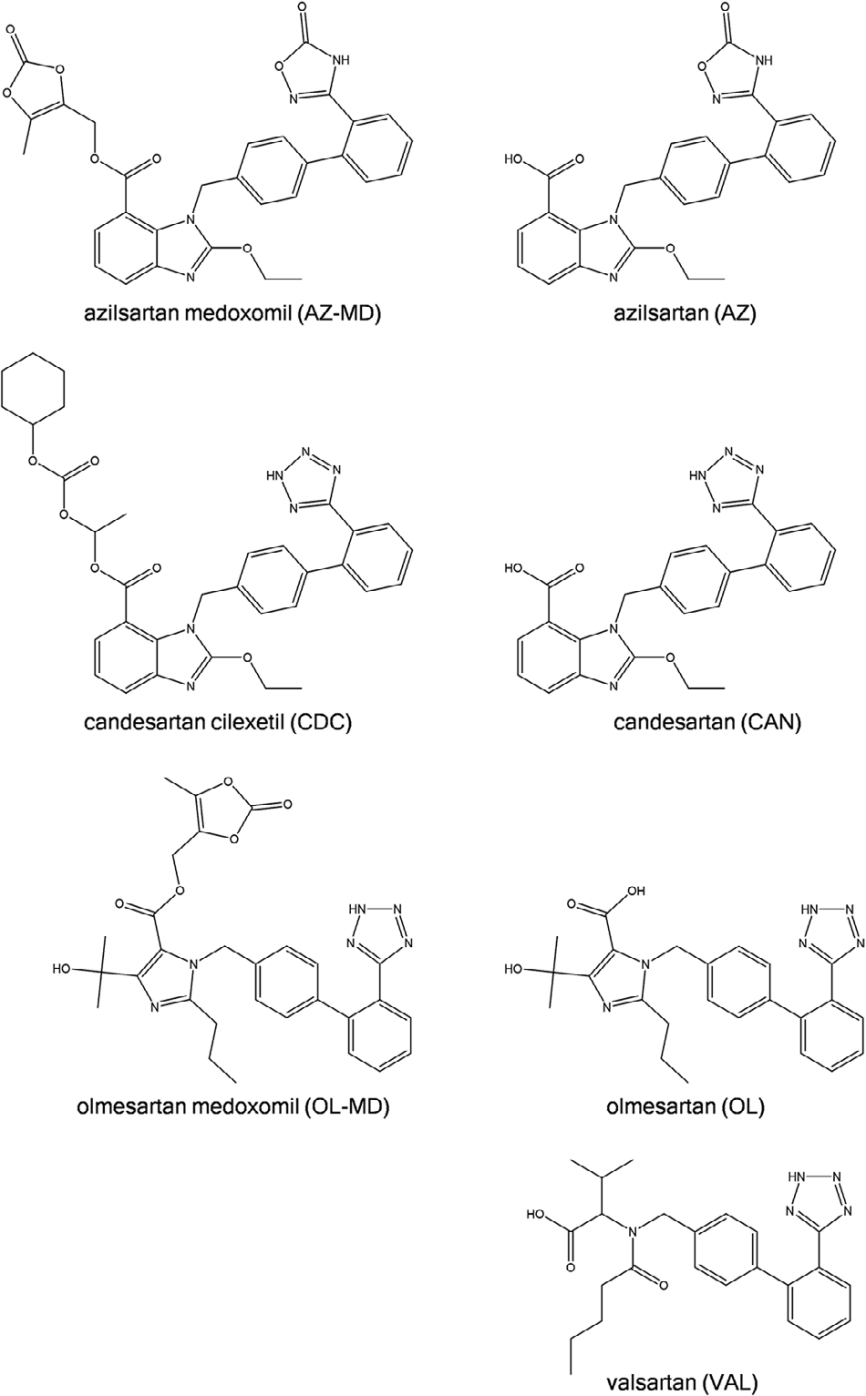
Structures of the selected sartans.

Eight different plants (cress, maize, pea, carrot, triticale, sorghum, rye, and barley) were cultivated hydroponically to investigate the uptake, translocation, and biotransformation of four widely used sartan drugs (for structures, see Figure 1), namely, azilsartan (AZ), olmesartan (OL), candesartan (CAN), and valsartan (VAL). In addition to the actual drugs, also the respective prodrug (candesartan cilexetil [CDC], azilsartan medoxomil [AZ‐MD], olmesartan medoxomil [OL‐MD]) was included in this study, as pollution of water, for example, through inappropriate disposal of tablets might also introduce these compounds into the environment [21]. This seems of particular importance, as, for example, in the case of CAN, the CDC proved stable in the window solutions for more than 30 days. These window solutions (i.e., solutions of the parent drug and/or prodrug) were used for comparative purposes. They were objected to daylight and analyzed after 2, 4, 8, and 12 days in order to explore the eventual generation of transformation products by the sole presence of light and water. Analysis of the plant extracts included HPLC separation with detection via high‐resolution MS and the measurement of drift‐times, allowing the calculation of CCS values. Results from these measurements were subjected to a search in an in‐house database (for details on the procedure and the database, please see Ref. [17]) in order to identify potential candidates for drug‐related metabolites formed in the plant organism. Such candidates were (in a second experiment) subjected to MS/MS measurements. Combining the information from the accurate mass determination, fragmentation in MS/MS experiments, data from chromatography (i.e., retention on an RP‐column), and CCS values (providing information on the “size” of the molecule) allowed the suggestion of molecular formulas and subsequently the suggestion of a tentative metabolite. Next to transformation products derived from changes in the molecular structure (hydroxylation, decarboxylation, dehydration, etc.), phase II metabolites formed by the addition of glucose and/or malonic acid, either to the parent drug or one of the derivatives listed before, were also found. Quite remarkable is the fact that in maize (as already reported in a previous study Ref. [22]), glucuronic acid conjugates were found for one of the drugs (CDC). A list of these tentative metabolites, found in extracts from different parts (roots, leaves, and in some cases, also stems) of eight edible plants, is provided in Tables S1–S4.

### 3.2 Uptake and metabolization of sartan drugs from actual WWTP effluents by cress

**FIGURE 2:**
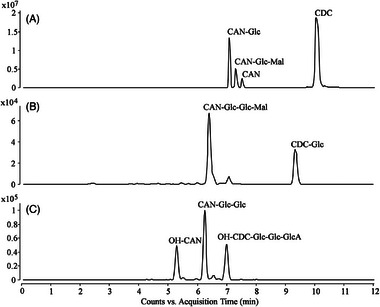
Extracted ion chromatograms (EICs) obtained for cress and maize root extracts that were grown in 10 mg L^−1^ candesartan cilexetil (CDC) solution: (A) EIC of CAN‐Glc, CAN‐Glc‐Mal, CAN, CDC; (B) EIC of CAN‐Glc‐Glc‐Mal, CDC‐Glc; (C) EIC of OH‐CAN, CAN‐Glc‐Glc, OH‐CDC‐Glc‐Glc‐GlcA. *Note*: for experimental conditions, see Section 2.

To evaluate the situation when plants are grown in waters from actual WWTPs, two samples from local WWTP effluents were drawn and analyzed with respect to the presence of the four selected sartans. In the first WWTP, OL and CAN were detected in concentrations of 0.9 and 4.1 µg L^−1^, respectively; AZ could be detected but not quantified—and no VAL was found. In the effluent water from the second WWTP, 5.8 µg L^−1^ of CAN and a relatively high concentration (23 µg L^−1^) of VAL were present, whereas none of the other two sartans were detected. The concentration range observed for the four sartans correlated well with data from the literature as well as results from WWTP effluent analyses previously performed in our lab. Subsequently, cress plants were grown hydroponically in the collected waters and treated in the same way (harvesting, separation of roots and upper part, extraction, etc.) as described in the experiments for the tentative identification of metabolites in the previous chapter. For the cress plants cultivated in the water from the WWTPs, the respective parent drugs could be clearly identified in root extracts, whereas in leaves only minor amounts (just above the LOD) were detected. Due to the fact that in the growing medium, the sartans were present in trace amounts only, hardly any metabolites could be detected in the plants. Only for CAN and VAL, glycosylated transformation products were found in the extracts from cress roots. Quantification of APIs in cress roots yielded concentrations of 3.1 ng g^−1^ for OL (cress cultivated in water from WWTP 1) and 10.4 ng g^−1^ for CAN and 14.4 ng g^−1^ for VAL (both in cress cultivated in water from WWTP 2). Extracted ion chromatograms for the relevant CAN‐related metabolites found in cress and pea can be found in Figure 2.

### 3.3 Comparison of targeted and nontargeted searches for drug metabolites in cress plants

Tentative drug metabolites in extracts from plant parts were so far detected by a more or less targeted approach (i.e., search in a database including potential phase I and phase II metabolites from the drugs included in this study). In a second set of experiments, the use of a nontargeted approach for detecting differences between “treated” and “nontreated” plants was employed. Five samples were taken from cress plants, which were grown in tap water and compared with five samples from cress plants grown in 10 mg L^−1^ AZ‐MD solution.

**FIGURE 3:**
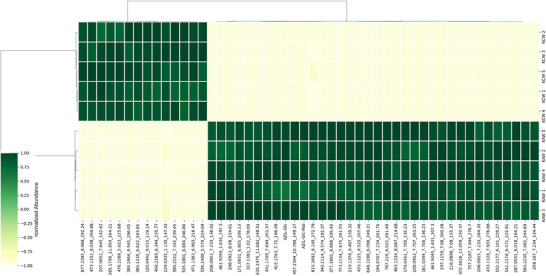
Hierarchical cluster heatmap displaying the 30 most significant features (based on *p*‐values resulting from *t*‐test).

For the comparison, features (signals representing a compound) were extracted from full MS scans of the plant extracts, and the resulting data were statistically analyzed. Figure 3 shows a heatmap displaying the most downregulated and the most upregulated compounds. The most upregulated features include two AZ metabolites, but most upregulated compounds, and of course all the downregulated ones, cannot directly be assigned to AZ‐MD and its metabolites. The presence of the pharmaceutical in the watering compartment clearly induced a significant overall change in the plant tissue, besides the uptake of the API and the formation of its metabolites.

**FIGURE 4:**
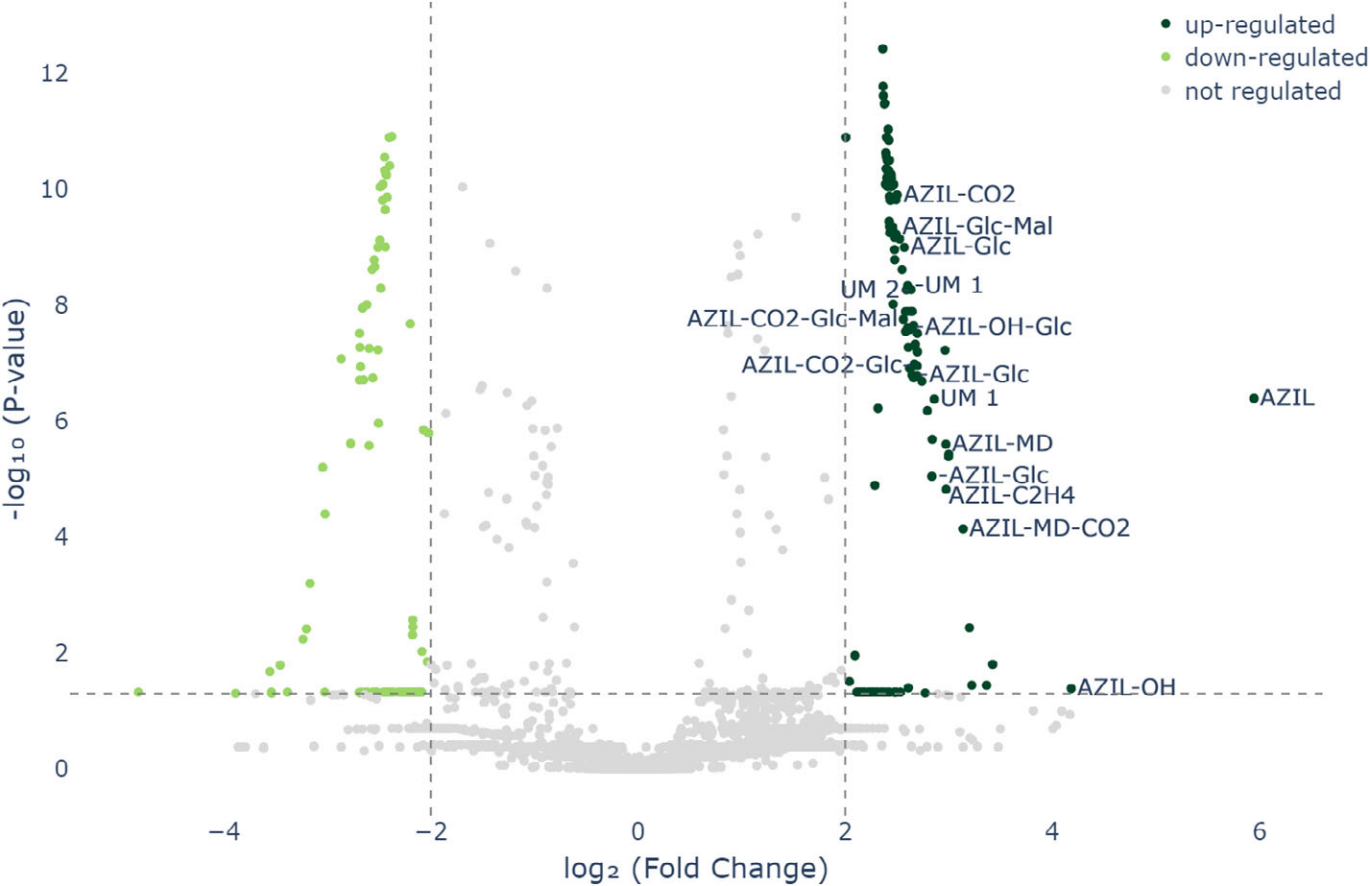
Volcano plot depicting significantly upregulated features on the top‐right side in dark green, whereas downregulated signals appear top‐left in light green.

In addition to the hierarchical cluster heatmap, a two‐dimensional volcano plot was created from *p*‐values and FCs to visualize which features were mainly responsible for the differences. Within the volcano plot (Figure 4), significantly upregulated features are depicted on the top‐right side (dark green), whereas downregulated signals appear on the top‐left (light green).

**FIGURE 5:**
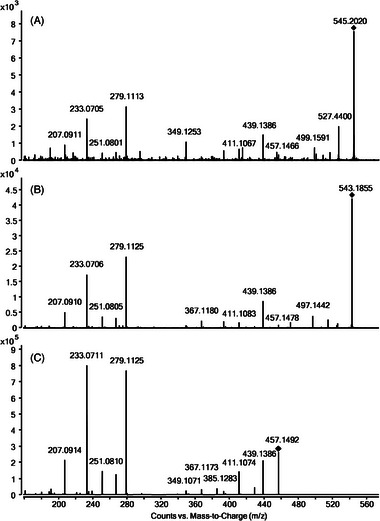
(A) Unknown metabolite (UM2) at CE 10 V; (B) unknown metabolite (UM1) at CE 10 V; (C) fragmentation pattern of azilsartan at CE 10 V.

In order to evaluate which upregulated features are directly related to AZ‐MD or AZ, MS/MS experiments were conducted, and the fragmentation patterns were compared with those of the unmetabolized molecule. Thereby, all previously detected metabolites were found in the API‐exposed plantlets. Moreover, next to nondrug‐related compounds, two additional substances were detected within the upregulated group with MS/MS data suggesting their origin from AZ. MS/MS spectra for these two substances (UM1, UM2) showing the characteristic fragmentation pattern of the parent drug are presented in Figure 5.

## 4 CONCLUDING REMARKS

In the present work, the uptake and transformation of four sartans by eight different edible plants were investigated. Thereby, more than 40 phase I and phase II metabolites, either derived from the parent drug or the prodrug, could be tentatively identified. As a proof of concept and to demonstrate that the selected approach would also be suitable for actual environmental analysis, cress plants were treated with effluents from WWTPs, and the parent drug as well as the most abundant metabolites could still be detected in the plant extracts. As using a targeted approach for analysis always includes the risk of overseeing analytes, nontargeted analysis was performed (comparing treated and untreated cress plants). Evaluation of results from the targeted “fast screening approach” developed in our lab and the nontargeted approach revealed the presence of two additional metabolites in AZ‐treated cress plants. Future investigations should put a closer focus on the large number of (so far) not identified features that were up‐ or downregulated upon the treatment of the plants with a drug‐containing solution.

## CONFLICT OF INTEREST STATEMENT

The authors have declared no conflict of interest.

## Supporting information

Supporting Information

## Data Availability

The data that support the findings of this study are available from the corresponding author upon reasonable request.
