# Supplementary material for: Uptake and metabolization of four sartan drugs by eight different plants: Targeted and untargeted analyses by HPLC‐drift‐tube‐ion‐mobility quadrupole time‐of‐flight mass spectrometry
Source: Electrophoresis. 2023 Nov 9;46(3-4):135–42. doi: 10.1002/elps.202300134 (PMC11865686; doi:10.1002/elps.202300134)

**uptake AND metabolization of four SARTAN drugs BY NINE DIFFERENT PLANTS: TARGETED AND UNTARGETED ANALYSIS by HPLC-drift-tube-ion-mobility quadrupole time-of-flight mass spectrometry**

Laura Zellner^1^, Thomas Schiefer^1^, Markus Himmelsbach^1^, Franz Mlynek^1^, and Christian W. Klampfl^1^*

^1^ Institute of Analytical and General Chemistry, Johannes Kepler University, Altenberger Strasse 69, 4040 Linz, Austria

**SUPPORTING INFORMATION**

Table S1: MRM transitions for QqQ analysis of drug related metabolites

| Compound Name | Fragmentor voltage [V] | Precursor ion [m/z] | Quantifier | | Qualifier | |
| --- | --- | --- | --- | --- | --- | --- |
|  |  |  | Product ion [m/z] | Collision energy [V] | Product ions [m/z] | Collision energy [V] |
| AZ | 75 | 457.0 | 233.1 | 12 | 279.2; 207.0; 180.2 | 0; 24; 32 |
| CAN | 114 | 441.2 | 263.1 | 5 | 423.2; 235.1; 207.1 | 5; 30; 40 |
| OL | 60 | 447.0 | 207.2 | 12 | 429.3; 149.0 | 0; 0 |
| VAL | 108 | 436.2 | 207.1 | 25 | 291.1; 235.1; 180.1 | 13; 13; 37 |

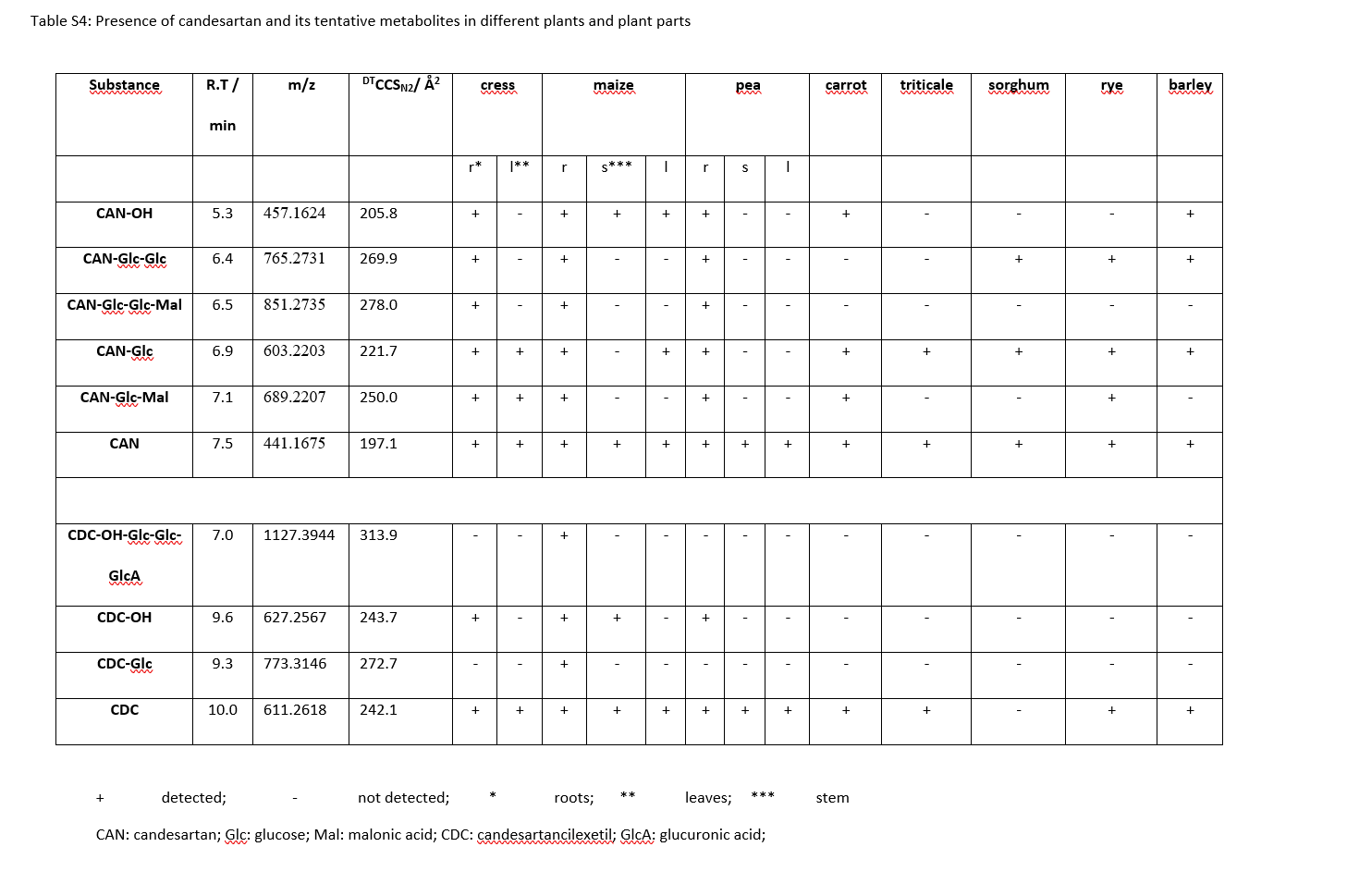

Supplement: Supplementary file 1 — Supporting Information [file ELPS-46--s001.docx]
